# Mental Health and Well-Being among Children of Public Safety Personnel in Canada

**DOI:** 10.3390/ijerph192114030

**Published:** 2022-10-28

**Authors:** Andrea M. Stelnicki, Laleh Jamshidi, Tamara L. Taillieu, R. Nicholas Carleton, Tracie O. Afifi

**Affiliations:** 1 Department of Psychology, University of Regina, Regina, SK S4S 0A2, Canada; 2 Department of Community Health Sciences, University of Manitoba, Winnipeg, MB R3E 0W3, Canada

**Keywords:** public safety personnel, families, mental health, child well-being

## Abstract

Public safety personnel (PSP) often experience stress due to their occupational demands that affect the family environment (e.g., work-family conflict, marital breakdown, disruption to home routines, and holidays). A substantial base of research has focused on the impact of PSP work on the marital relationship, but fewer studies have focused specifically on children’s functioning within PSP families. The current study investigated mental health, well-being, and functioning among children of PSP in Canada, as reported by PSP. Data were collected between 2016 and 2017 as part of a large pan-Canadian study of PSP. Participants (*n* = 2092; 72.5% women) were PSP parents who responded to questions about their 4- to 17-year-old children. Overall, a substantial proportion of PSP parents reported their children have at least some difficulties with sadness (15.4%), worries and fear (22.0%), disobedience or anger (22.0%), attention (21.0%), and friendships (11.4%). Firefighters reported the fewest problems among their children compared to other PSP groups. Almost 40% of participants indicated that their child’s problems were related to their work as a PSP. The results highlight the need to find ways to identify children that are struggling and provide support to those families. Organizations and PSP leadership should develop and prioritize efforts to support families of PSP members, with the likely outcome of enhancing PSP member well-being.

## 1. Introduction

Public safety personnel (PSP; e.g., border services officers, correctional workers, firefighters, paramedics, police, public safety communications officials) experience stressors related to their work, such as shift work, unpredictable overtime hours, insufficient support from leadership, threats to personal safety, and exposure to potentially psychologically traumatic events (PPTEs) [1,2]. The diverse stressors can impact the ability of PSP to do their job effectively, and can impact their home environment and their family. Indeed, the demands of PSP work can simultaneously lead to work–family conflict (e.g., job interfering with family life, no time for family activities) and emotional exhaustion (e.g., emotionally drained from work) [3]. Shift work can have a substantial impact on work-family life, such as disruption to home routines, holiday time, and changes in family roles [4]. Having insufficient time available to spend with family and friends because of work is reported as particularly stressful for PSP [1]. Spouses of PSP indicate that inconsistent work schedules, shift changes, and job commitments competing with family commitments are also all significant stressors that can impact their family [1,5].

Canadian PSP have reported negative familial effects due to occupational demands, including marital breakdown, relationship problems with their children, and increased stress at home [6]. There is also substantial evidence that PSP work can negatively impact marital relationships and the life experiences of PSP spouses [2,7,8,9]; however, fewer studies have focused specifically on children’s functioning within PSP families. For example, children of PSP report both advantages and disadvantages of their parents’ work schedules (e.g., shiftwork); children report getting to spend a lot of time with their parent during off-shift days, but also report their parents inevitably miss family events because of work [4,10]. Exposure to news and media increase children’s fears about their parents’ jobs [10]. Adolescent children of police officers experience conflicted feelings between their loyalty and pride in their parent’s work, and their feelings of rejection by peers who hold negative attitudes about police officers and authority figures [5]. Left unaddressed, the conflicts may result in disruptions to family and peer systems that negatively impact the child’s mental health or use of healthy coping strategies.

Most child-centric research has focused on secondary or vicarious traumatic stress (i.e., stress following indirect exposure to a PPTE experienced by their parent; [11]). Children are at risk of psychopathology when their parents are exposed to violent events and other PPTEs, independent of the child’s direct exposure [12,13,14]. Research following the World Trade Center attacks indicated children of paramedics experienced vicarious traumatic stress symptoms, such as repetitive traumatic play, separation anxiety, difficulties with sleep, and behaviour problems [15]. Children of paramedics who responded to the World Trade Center attacks experienced more problems than children of police officers and firefighters, suggesting differences may exist among PSP groups [16]. Many more children (18.9%) from families with a paramedic met criteria for posttraumatic stress disorder (PTSD) than did children from families with police officers (10.6%), firefighters (5.6%), or no PSP (10.1%) [16]. Similarly, children with paramedic family members had higher prevalence of probable major depressive disorder than children in families with police officers, firefighters, or no PSP following the World Trade Center attacks [17]. Many police officers (20%) who were on scene at the World Trade Center reported significant subsequent behavioural problems in their children, even four years later [18]. High prevalence of PTSD, emotional symptoms, and hyperactivity/inattention were also reported in youth of first responders following the Boston Marathon attack, even after adjusting for children’s direct exposure to the attack [19]. Overall, problems impacting children exposed to secondary traumatic stress appear potentially long-lasting without appropriate recognition and intervention.

### Current Study

The current study investigated mental health and well-being among children of Canadian PSP and contributes to the very limited research literature regarding the functioning of children from PSP families. Specifically, the study addresses six research questions: (1) What is the prevalence of unhappiness, worry, anger, attention problems, few friendships, and sensitivity to others among children of PSP?; (2) Does the prevalence of unhappiness, worry, anger, attention problems, few friendships, and sensitivity to others vary by type of PSP; (3) Is children’s emotional well-being related to parents’ work as a PSP; (4) Does children’s poor emotional well-being relate to parents’ work vary by type of PSP; (5) What is the prevalence of parent-reported mental disorder among children of PSP; and (6) Does the prevalence of parent-reported mental disorder among children vary by type of PSP?

## 2. Materials and Methods

### 2.1. Procedure and Sample

Data were collected as part of a bilingual pan-Canadian web-based self-report survey of PSP between September 2016 and January 2017. The survey was collaboratively designed by researchers from the University of Regina and the Public Safety Steering Committee (PSSC) of the Canadian Institute for Public Safety Research and Treatment (CIPSRT). The survey link was distributed by email to provincial and municipal PSP agencies and by the PSSC. Additional information regarding participant recruitment is available in a previous publication [20]. Participation was voluntary and anonymous. The study was approved by the University of Regina Institutional Research Ethics Board (File #2016-107).

PSP were grouped by category: municipal/provincial police; Royal Canadian Mounted Police (RCMP); correctional workers; firefighters; paramedics; and public safety communicators (e.g., call centre operators/dispatchers). A total of *n* = 8520 PSP began the survey. Several participants (*n* = 2092; 72.5% women) reported having children aged 4- through 17-years-old and completed relevant questions for the current analyses. PSP participants who reported having adult children only were omitted from the analyses. Further, participants did not provide information regarding their adult children if they had both adult children and children under 18-years-old. Participants reported on a total of 3726 children. Families predominantly reported having one (*n* = 836, 25.1%) or two children (*n* = 955, 28.7%) ages 4 to 17 years, and provided well-being and functioning about each child individually.

### 2.2. Measures

Sociodemographic variables. Several sociodemographic variables for PSP and children were included in the analyses. Sociodemographic variables for PSP included sex (male or female), age (18 to 29 years, 30 to 39 years, 40 to 49 years, 50 to 59 years, or 60 years and older), province of work (Western Canada [British Columbia, Alberta, Manitoba], Eastern Canada [Ontario, Quebec], Atlantic Canada [Newfoundland & Labrador, New Brunswick, Prince Edward Island, Nova Scotia], and Northern Territories [Yukon, Northwest Territories, Nunavut]), marital status (married/common-law, remarried, separated/divorced/widowed, or single), ethnicity (white or other), highest level of educational attainment (high school or less, some post-secondary [less than 4-year college/university program], or university degree/4-year college or higher), PSP type (public safety communicators, correctional workers, firefighters, paramedics, municipal/provincial police, or RCMP), and total years of service (under 4 years, 4 to 9 years, 10 to 15 years, or more than 15 years). Children’s sociodemographic variables included age, sex (male or female), and residence (in PSP households full time, in PSP households part time, or outside of PSP households full time).

Children’s well-being. Children’s well-being was assessed using five author-created items assessing unhappiness (i.e., “appears unhappy”), worry (i.e., “appears worried and/or fearful”), disobedience/anger (i.e., “is disobedient and/or easily distracted”), attention problems (i.e., “is always ‘on the go’ and/or is easily distracted”), friendships (i.e., “appears to have few friends”), and sensitivity to others (i.e., “is sensitive to other peoples’ feelings and need”). The items were created based on brief screening questions commonly used in clinical practice to indicate whether further assessment for mood, anxiety, attention, or other disorders is warranted. PSP were asked to rate whether each statement was characteristic of their child (1 = not at all; 5 = entirely). PSP were asked to report whether they believe the child’s emotions and/or behaviours interfered with the child’s everyday life in four areas: at home, social life, school, and leisure activities. Parent PSP responded using a five-point scale (1 = not at all; 5 = extremely).

Relation to PSP work. Parent PSP were asked whether they believed the child’s emotional and/or behavioural problems were related to their work or the consequences of their work using a single item. Response options were “no”, “yes”, or “at least in part.”

Mental health diagnosis. Mental health diagnosis was assessed using a parent self-report question, “have any of your children ever been diagnosed with a mental health disorder?”, and information was provided for each child as applicable.

### 2.3. Statistical Analyses

First, several cross tabulations were performed to examine the prevalence of child’s well-being variables responses, the frequencies of child’s well-being variables among PSP type categories, the association between the child’s emotions and/or behaviors and parent’s work as PSP, prevalence of mental health disorder diagnosis among children of PSP, and prevalence of child’s impairment in different domains. A series of logistic regression models were used to determine if the prevalence of parents who indicated that each well-being variable was “very characteristic” or “entirely characteristic” of their child varied by PSP group. Similarly, differences in parent-reported mental disorders among children by PSP group were analyzed by a series of logistic regression models. Logistic regression models were adjusted for the age of children of PSP.

## 3. Results

PSP respondent characteristics and sociodemographic categories are shown in Table 1. PSP respondents were mainly women (72.5%) and were 40 to 49 years old (56.8%). PSP predominantly worked in Western Canada (51.0%) and Eastern Canada (35.0%). Most participants were municipal/provincial police (29.5%) or RCMP (25.7%), with most PSP reporting over 15 years of service (64.0%). PSP primarily reported being married or in common-law relationships (83.0%). Considerable proportions of PSP had some post-secondary (55.8%) or a university (36.4%) education. There were similar proportions of male (50.3%) and female (49.4%) children, and children were mainly aged 8 to 12 years old (37.5%). Most children resided full time in the PSP households (81.3%).

Frequencies of child well-being markers (as reported by their parents) are presented in Table 2. Among the well-being markers, participants indicated that 15.4% of children were “somewhat” (9.0%), “very” (4.9%), or “entirely” (1.5%) unhappy. Participants also largely indicated that their children did not appear worried and/or fearful (44.2%); however, a larger portion of participants indicated their children were “a little bit” (33.2%), “somewhat” (13.4%), “very” (6.8%), or “entirely” (1.8%) worried and/or fearful. Similar proportions of participants indicated their children were “a little bit” (28.4%), “somewhat” (13.2%), “very” (6.8%), or “entirely” (2.0%) disobedient and/or easily angered. For the attention marker, 27.8% of participants indicated that “always ‘on the go’ and/or is easily distracted” characterized their child “a little bit”, whereas 38.5% said this described their child “somewhat” (17.5%), “very” (14.4%), or “entirely” (6.6%). Participants reported their children did not appear to have few friends (58.6%). A large percentage of participants indicated “is sensitive to other peoples’ feelings and need” was very characteristic (33.4%) or entirely characteristic (22.1%) of their child.

Parent reports of child well-being indicators are organized by PSP type and presented in Table 3. Few differences were observed across PSP categories. No differences were observed between children of different PSP groups for “appears unhappy”, “is always ‘on the go’ and/or is easily distracted”, and “is sensitive to other people’s feelings and needs.” Statistically significant differences were found for some variables. For “appears worried and/or fearful”, firefighters and municipal/provincial police reported the lowest prevalence, which were statistically significantly lower prevalence than other groups. Municipal/provincial police reported the lowest prevalence for “disobedient and/or easily becomes angry”. Firefighters reported lower prevalence than other groups for their children on the variables of “appears to have few friends” and “disobedient and/or easily becomes angry”. Children of RCMP were rated to have the highest prevalence of children who “appear to have few friends” compared to other groups.

Associations between children’s emotions and/or behaviours and parent’s work as PSP are presented in Table 4. Most parents (60.6%) did not attribute their children’s emotions and/or behaviours to their work, and 39.4% of PSP indicated their work was definitely or at least in part associated with their children’s emotional and/or behavioural problems. More RCMP parents (48.6%; AOR = 1.42, 95% CI = 1.12–1.79) reported their children’s problems were related to their work than did the other PSP categories, and public safety communicators (31.9%; AOR = 0.70, 95% CI = 0.46–1.08) and municipal/provincial police (33.5%; AOR = 0.75, 95% CI = 0.59–0.95) were least likely to report that their children’s problems were related to their work. Table 4 also shows the parent-reported prevalence of parent-reported mental disorder diagnoses among children. Thirteen percent of parents reported that their children have received a mental disorder diagnosis. There were some statistically significant associations between child’s mental disorder diagnosis and PSP type, with paramedics reporting the highest prevalence (16.9%; AOR = 1.43, 95% CI = 0.96–2.11) of mental disorders within their children, followed by public safety communicators (16.4%; AOR = 1.28, 95% CI = 0.72–2.27).

## 4. Discussion

The current study is the first to provide an indication of the well-being of children aged 4–17 years of a diverse sample of PSP in Canada, as reported by their parents. Overall, a substantial proportion of PSP parents reported their children have at least some difficulties with sadness (15.4%), worries and fear (22.0%), disobedience or anger (22.0%), attention (21.0%), and friendships (11.4%). The prevalence of worry and difficulties with friendships showed some differences across PSP types, with children of firefighters exhibiting the fewest number of problems in these areas. Municipal/provincial police were least likely to report that their children have difficulty with disobeying and anger. Children of RCMP had higher prevalence of friendship problems than other PSP groups.

There were few differences in children’s well-being across PSP categories. RCMP parents were more likely to report believing their children’s problems were entirely or partially related to their work. RCMP often have different occupational demands compared to other PSP, including frequent relocation (often to rural locations) and working alone instead of with a partner. Vocational factors specific to RCMP may lead to a lack of perceived social support by the RCMP parent, which has been consistently associated with increased stress [21]. Frequent relocations can increase stress for the family and can result in school disruptions for the child [22,23,24]. Children relocating to a new school can take up to 6 months to fully adjust to new school expectations and peer groups [25]. As such, relocation may help explain why RCMP parents were more likely to report their work impacts their children more than other PSP groups. Additionally, relocation could also explain why children of RCMP parents were more likely to report that their children had fewer friends than other groups. PSP members are most likely to turn to informal supports when seeking support (e.g., spouses, friends) [26]; if their children follow the same pattern, children of RCMP officers may not have the informal support base to turn to compared to other PSP groups. No longitudinal research has yet focused on RCMP (or other Canadian PSP groups) and occupational impacts on their families.

A substantial portion of PSP parents (i.e., 50.6–66.9%) did not attribute their children’s problems to PSP work or consequences of PSP work; however, almost half (48.6%) of RCMP parents and over 40% of correctional worker and paramedic parents did believe that their work may be contributing to their children’s problems. RCMP were more likely to report that their work contributed to their children’s problems. Relatedly, a small, but significant proportion of PSP parents indicated that their children have been diagnosed with a mental disorder (i.e., 10.8–16.3%), and paramedics were most likely to report previous diagnoses in their children. The results appear to be similar to Canadian estimates that 10–20% of children aged 4–17 experience a mental disorder at any given time [27,28,29]. Nonetheless, only 1 in 5 children who need mental health services actually receive such services [28]. Measures that can detect for subclinical mental disorder symptoms should be used in future research with PSP families, and statistical comparisons between PSP families and the general population of young people should be tested.

A large proportion of the sample (55.5%) reported their children demonstrated prosocial behaviour (i.e., “is sensitive to other people’s feelings and needs”), which may suggest the presence of social strengths. Social competence appears to be a protective factor against the development of problems, with lower social competence predicting increased internalizing and externalizing behaviours across childhood [30,31,32]. Adaptive functions, such as self-control in social situations, also protect against the development of problems [33,34]. For example, early acting out may disrupt the development of social skills and successful relationships as children get older [32,35]; as such, whether prosocial behaviour is a precursor to, or consequence of, well-developed adaptive skills remains unclear.

### Limitations and Future Directions

The current study has several limitations that offer directions for future research. First, although the sample of participants was diverse, the sampling methods used pose a limitation to the generalization of the results to all PSP in Canada, as described previously [20]. Unlike other large-scale PSP research [20], the sample who chose to answer the relevant questions related to family in the current study was predominantly female (72.5%). Additionally, the sample was self-selected, and it is not possible to calculate a true response rate; Carleton et al. [20] estimates that 5% of PSP in Canada responded to the larger survey, but it is difficult to say with certainty what proportion of the PSP population was reached via recruitment methods. We also do not have specific estimates of mental health rates within the general population group to determine if our rates are higher than youth who do not live in PSP families.

Second, the length and purpose of the original survey (i.e., PSP mental health and associated factors) prohibited inclusion of in-depth questions about children’s well-being and functioning. The indicators of well-being that were chosen for inclusion are far from diagnostic and provide only an estimate of children’s well-being. Verification of parent-reported child mental disorder diagnoses was not possible, and the source of diagnosis (e.g., psychologist, family doctor, psychiatrist) was not asked. Future research with children of PSP should use longer, reliable, and valid measures to estimate problematic behaviour, well-being, as well as screen for mental disorders to enhance clinical and research applications [36]. Comparisons across children in different developmental stages would also provide important insights for intervention options.

Third, children’s well-being was reported by only one parent with no corroborating information. Parents may demonstrate inconsistent ratings of their children’s internalising problems and moderately agree on externalizing and total problems, as has been found in military families [37]. Parent ratings of problematic behaviour are often discordant from the child’s self-rating [37,38], and fathers tend to rate children to have fewer problems than mothers [39]. Further, if parents are experiencing their own stress, as many PSP do [1,20], they may not be accurate reporters of children’s problems [40].

Future research should also include PSP mental health status, since children who have parents with mental disorders are more likely to have developmental problems or their own mental disorder diagnosis [41,42,43]. Additionally, it is important to include other important sociodemographic variables in our models to examine the impact of marital status, parent education, and geographic region, which are important predictors of PSP mental health symptom endorsement [20]. It would also be interesting to look at the impact of number of children in the household and the reciprocal relationships between each family members’ well-being. Researchers should aim to include behaviour ratings from all family members, in combination with clinical verification, to obtain the best overall picture of the family’s functioning.

Lastly, we were unable to analyze whether the children reported on were born before or after the PSP started their career and the cross-sectional data does not allow for causal claims. Longitudinal, mixed-methods studies with PSP families could clarify whether the development of problems in children are associated with the PSP parents’ work obligations and occupational stress.

## 5. Conclusions

The current study contributes to our nascent understanding the functioning of children from Canadian PSP families. A substantial and important portion of participants reported indicators of problematic behaviour among their children. Moving forward, it will be important to identify factors that are associated with poorer outcomes among PSP families. Almost half of RCMP and 40% of paramedic and public safety communicator parents reported their children’s problems were related (at least in part) to their work. This finding has important implications, as some PSP groups may benefit from targeted family programming to support their children.

The limited research with Canadian PSP families and the current study provides preliminary evidence to support additional research programs examining this population, particularly aimed at children with PSP parents. Many PSP families reported that their children were functioning well, but many reported issues that cannot be ignored. There are benefits to providing support to PSP families that would likely result in enhanced well-being of both family functioning and PSP members. The large percentage of families who are experiencing issues with their children’s emotional and social development suggest that organizations and PSP leadership should prioritize efforts to support families of PSP members.

## Figures and Tables

**Table 1:** Sociodemographic distribution among public safety personnel and their children.

Demographic Variables	% (*n*)
PSP Demographic Variables	
**Sex**	
Female	72.5 (1514)
Male	27.5 (575)
**Age**	
18–29	0.8 (17)
30–39	24.2 (505)
40–49	56.8 (1186)
50–59	17.7 (369)
60 and older	0.5 (10)
**Provincial Region**	
Western Canada (British Columbia, Alberta, Saskatchewan, Manitoba)	51.0 (1061)
Eastern Canada (Ontario, Quebec)	35.0 (728)
Atlantic Canada (Prince Edward Island, Nova Scotia, New Brunswick, Newfoundland)	12.9 (268)
Northern Territories (Yukon, Northwest Territories, Nunavut)	1.2 (25)
**Marital Status**	
Married/Common-law	83.0 (1730)
Single	1.9 (40)
Separated/Divorced/Widowed	11.0 (229)
Re-married	4.1 (86)
**Ethnicity**	
White	90.9 (1898)
Other	9.1 (190)
**Education**	
High school or less	7.8 (159)
Some post-secondary (less than 4-year college/university program)	55.8 (1140)
University degree/4-year college or higher	36.4 (743)
**PSP Type**	
Public Safety Communicators (e.g., Call Center Operators/Dispatchers)	3.7 (78)
Correctional workers	11.8 (246)
Firefighters	14.2 (298)
Paramedics	11.1 (232)
Municipal/Provincial Police	29.5 (617)
RCMP	25.7 (537)
**Years of Service**	
Under 4 years	1.3 (28)
4 to 9 years	8.9 (186)
10 to 15 years	25.8 (540)
More than 15 years	64.0 (1338)
**Children’s Demographic Variables**	
**Sex**	
Male	50.3 (1828)
Female	49.4 (1794)
**Residence**	
In PSP households (full time)	81.3 (2924)
In PSP households (part time)	15.0 (538)
Outside of PSP households (full time)	3.6 (128)
**Age**	
4–7	27.2 (1015)
8–12	37.5 (1398)
13–17	35.2 (1312)

**Table 2:** Prevalence of well-being variables among children of public safety personnel.

	Not at All Characteristic of My Child	A Little Bit Characteristic of My Child	Somewhat Characteristic of My Child	Very Characteristic of My Child	Entirely Characteristic of My Child
	% (*n*)	% (*n*)	% (*n*)	% (*n*)	% (*n*)
Appears unhappy	58.2 (2116)	25.6 (932)	9.0 (328)	4.9 (179)	1.5 (56)
Appears worried and/or fearful	44.2 (1604)	33.2 (1205)	13.4 (485)	6.8 (247)	1.8 (66)
Is disobedient and/or easily becomes angry	48.9 (1775)	28.4 (1032)	13.2 (481)	6.8 (248)	2.0 (73)
Is always “on the go” and/or is easily distracted	33.1 (1203)	27.8 (1008)	17.5 (635)	14.4 (523)	6.6 (239)
Appears to have few friends	58.6 (2128)	18.8 (684)	10.6 (384)	7.6 (277)	3.8 (137)
Is sensitive to other people’s feelings and needs	8.2 (299)	15.6 (568)	20.0 (726)	33.4 (1213)	22.1 (802)

**Table 3:** Prevalence of Children Described as Very Characteristic or Entirely Characteristic for Well-Being Variables by Public Safety Personnel Type and Relationship Between Well-Being Variables and Public Safety Personnel Type.

	Correctional Workers	Public Safety Communicators (e.g., Call Center Operators/ Dispatchers)	Firefighters	Paramedics	Municipal/ Provincial Police	RCMP
	% (*n*) AOR (95% CI)	% (*n*) AOR (95% CI)	% (*n*) AOR (95% CI)	% (*n*) AOR (95% CI)	% (*n*) AOR (95% CI)	% (*n*) AOR (95% CI)
Appears unhappy	6.3 (27) 1	5.8 (7) 0.99 (0.42,2.33)	6.8 (36) 1.13 (0.67,1.90)	5.9 (23) 1.03 (0.58,1.83)	6.6 (71) 1.14 (0.72,1.81)	6.9 (64) 1.20 (0.75,1.91)
Appears worried and/or fearful	10.6 (45) 1	10.1 (12) 1.03 (0.52,2.02) ^b^	5.5 (29) 0.51 (0.32,0.83) ^a^	10.5 (41) 1.12 (0.71,1.75) ^b^	10.5 (41) 0.68 (0.46,1.00) ^a^	6.8 (73) 1.03 (0.70,1.50) ^b^
Is disobedient and/or easily becomes angry	12.6 (54) 1	13.3 (16) 1.06 (0.58,1.93) ^a^	8.0 (42) 0.60 * (0.39,0.91) ^a,b^	10.2 (40) 0.78 (0.51,1.21) ^a^	6.3 (67) 0.46 *** (0.31,0.67) ^b^	9.2 (86) 0.70 (0.48,1.00) ^a^
Is always “on the go” and/or is easily distracted	23.7 (101) 1	24.2 (29) 0.99 (0.61,1.59)	21.8 (115) 0.88 (0.65,1.19)	21.5 (84) 0.83 (0.60,1.16)	18.3 (197) 0.69 ** (0.53,0.91)	21.7 (202) 0.85 (0.64,1.11)
Appears to have few friends	10.5 (45) 1	14.2 (17) 1.49 (0.82,2.72) ^a^	8.7 (46) 0.84 (0.54,1.30) ^a^	9.5 (37) 0.96 (0.61,1.53) ^a^	10.6 (114) 1.08 (0.75,1.55) ^a^	14.5 (135) 1.55 * (1.08,2.23) ^b^
Is sensitive to other people’s feelings and needs	53.5 (228) 1	60.0 (72) 1.31 (0.87,1.98)	56.5 (298) 1.13 (0.88,1.47)	55.2 (216) 1.08 (0.82,1.42)	57.9 (621) 1.20 (0.96,1.51)	54.2 (506) 1.04 (0.82,1.31)

Note: AOR: Adjusted Odds Ratio for Children’s age; Different lettered superscripts indicate categories of public safety officers that are significantly different from one another at *p* ≤ 0.05 (e.g., groups with ^a^ are statistically significantly different from groups with ^b^). * *p* < 0.05, ** *p* < 0.01, *** *p* < 0.001 indicate statistically significant differences from reference group (i.e., Correctional Workers).

**Table 4:** Association between the child’s emotions and/or behaviours and parent’s work as public safety personnel and prevalence of mental health disorder diagnosis among children of public safety personnel.

	Total	Correctional Workers		Public Safety Communicators (e.g., Call Center Operators/Dispatchers)		Firefighters		Paramedics		Municipal/ Provincial Police		RCMP	
	% (*n*)	% (*n*)	AOR (95% CI)	% (*n*)	AOR (95% CI)	% (*n*)	AOR (95% CI)	% (*n*)	AOR (95% CI)	% (*n*)	AOR (95% CI)	% (*n*)	AOR (95% CI)
	***Do you think your child’s emotional and/or behavioural problems are related to your work or the consequences of your work?***												
Yes/ At least in part	39.4 (1361)	40.6 (173)	1	31.9 (38)	0.70 (0.46,1.08) ^a^	35.1 (183)	0.80 (0.61,1.04) ^a^	40.5 (157)	1.02 (0.77,1.35) ^a^	33.5 (358)	0.75 * (0.59,0.95) ^b^	48.6 (452)	1.42 ** (1.12,1.79) ^c^
No	60.6 (2094)	59.4 (253)		68.1 (81)		64.9 (339)		59.5 (231)		66.5 (712)		51.4 (478)	
	***Have any of your children ever been diagnosed with a mental disorder?***												
Yes	13.0 (444)	14.9 (62)	1	16.4 (19)	1.28 (0.72,2.27) ^a,b^	11.2 (57)	0.75 (0.51,1.12) ^a^	16.9 (65)	1.43 (0.96,2.11) ^b^	12.0 (128)	0.90 (0.65,1.26) ^a^	12.2 (113)	0.94 (0.66,1.32) ^a^
No	87.0 (2970)	85.1 (354)		83.6 (97)		88.8 (453)		83.1 (320)		88.0 (935)		87.8 (811)	

Note: AOR: Adjusted Odds Ratio for Children’s age; Different lettered superscripts indicate categories of public safety officers that are significantly different from one another at *p* ≤ 0.05 (e.g., groups with ^a^ are statistically significantly different from groups with ^b^ or ^c^). * *p* < 0.05, ** *p* < 0.01—Statistically significant.

## Data Availability

The datasets generated for this study will not be made publicly available as data security procedures were made explicit by all parties prior to collection. Output from analyses with de-identified data may be available.
